# The Psychological Consequences of COVID-19 Fear and the Moderator Effects of Individuals’ Underlying Illness and Witnessing Infected Friends and Family

**DOI:** 10.3390/ijerph18041836

**Published:** 2021-02-13

**Authors:** Orhan Koçak, Ömer Erdem Koçak, Mustafa Z. Younis

**Affiliations:** 1 Faculty of Health Sciences, İstanbul University—Cerrahpaşa, İstanbul 34500, Turkey; 2 Faculty of Business and Managerial Sciences, Istanbul Medipol University, İstanbul 34810, Turkey; oekocak@medipol.edu.tr; 3 College of Health Sciences, Jackson State University, Jackson, MS 39217, USA; younis99@gmail.com

**Keywords:** pandemic, COVID-19, fear, depression, anxiety, stress

## Abstract

The COVID-19 virus has become a fearful epidemic for people all over the world. In Turkey, long quarantine periods and curfews have increased both physical and psychological problems. Due to the rapid spread and substantial impact of the COVID-19 virus, different psychological effects were observed among different segments of society, such as among young people, elderly people, and active workers. Because of fear caused by the COVID-19 virus, it is thought that depression, stress, and anxiety levels have increased. It is estimated that there are more psychological issues for people with poor health and others whose friends or family became ill or have died because of COVID-19. To explore and test the situation mentioned above, we conducted a cross-sectional study in Turkey with 3287 participants above 16 years old. We measured COVID-19 fear, along with anxiety, stress, and depression levels (DASS21) and demographics. Firstly, we tested whether COVID-19 fear predicts stress, anxiety, and depression. Secondly, we investigated if the effect of COVID-19 fear is stronger for those who have underlying illness and for those whose friends or family became ill or have died because of COVID-19. The results showed that women and 16–25 years old youths have higher COVID-19-related fear, anxiety, depression, and stress. Furthermore, we found a significant relationship between COVID-19 fear and stress, anxiety, and depression, as well as significant moderation effects of having an underlying illness and having friends or family who were infected or have died. These results show the importance of implementing specific implementations, particularly for vulnerable groups, to minimize the psychological problems that may arise with the pandemic.

## 1. Introduction

In December 2019, the first COVID-19 virus was reported in Wuhan, China. Since then, the virus has spread to South East Asia, Europe and nearly every country in the world a year later. The World Health Organization legally recognized the COVID-19 virus as a pandemic in March 2020 [1]. The number of cases in the world is 75.5 million as of December 20th; of these, 42.5 million have recovered, while 1.67 million have died. In Turkey, the number of cases is 2 million; 1.72 million have recovered, 17,610 have died [2].

Societies experience psychological traumas and physical problems both during and after a pandemic [3]. The behavior prompted by the COVID-19 outbreak have resulted in life constraints that we did not believe we would be subjected to before the pandemic [4]. Individuals are not allowed to meet with their friends, and cannot perform daily activities that they used to do before, feeling more isolated than they had before [5]. COVID-19 has triggered multiple psychological issues, including anxiety, depression, and sleep disturbances, similar to previous pandemics. Social alienation, physical distance, and quarantines have caused people to feel totally isolated and increased their depression [6]. While the new normal life caused by COVID-19 has changed many of our habits and behaviors, it has also negatively affected our psychology. In addition to the rapidly increasing number of cases in many countries in recent weeks, the uncertainty caused by not knowing when the epidemic will end increases anxiety and has triggered mental problems.

The presence of the risk factors listed below increases the possibility of an individual to be diagnosed with major depression, post-traumatic stress disorder, and/or anxiety disorder [7]. Those factors are (i) having been diagnosed with a mental disorder or chronic physical illness before the COVID-19 outbreak (treated or still being treated), (ii) being diagnosed with COVID-19, being treated for COVID-19, or being quarantined, (iii) death of a relative or friend from COVID-19, (iv) infection of a relative or friend with the COVID-19 virus, (v) exposure to intense stress during this period, and (vi) serious economic loss, job loss, or bankruptcy caused by the pandemic [7].

The hypothesis of this study is based on a work by Işıklı [7], who suggested that there are psychological implications caused by the fear of COVID-19, and that risks such as illness, virus transmission, or virus death will further increase these psychological consequences. Therefore, this study attempted to understand the effects of COVID-19 fear levels on depression, anxiety, and stress levels of people during the COVID-19 pandemic. Moreover, moderation analyses were made to measure the effects of COVID-19 fear on depression, anxiety, and stress according to people’s health conditions and their family’s or friends’ infection status. Research has shown that the fear of COVID-19 has a negative impact on people’s psychology. This effect varies according to people’s health condition and whether there are infected or dead among their families and relatives.

### 1.1. COVID-19 Fear of Individuals

In the first weeks, countries did not know how to combat the COVID-19, and the WHO declared COVID-19 as a pandemic on 18 March 2020, after the deaths of 8000 people worldwide [8,9,10,11,12]. The first COVID-19 case was declared by the Ministry of Health in Turkey on 12 March 2020. Although the cases decreased in the summer period, the number of cases increased again in September. As in other European countries, in Turkey, the second wave started in October and November. The number of people infected was more than 2 million and the number of people who died was more than 20,000 as of 20 December 2020 [13].

Schools and universities have remained closed, while working hours in private workplaces, public institutions, restaurants, and entertainment places were restricted. To support reducing the number of infected people, a “Stay at Home” campaign was launched. When the psychological consequences of the call to “Stay at Home” for isolation during the pandemic are examined, it is understood that this practice, critical for protecting physical health, has negative psychological and economic consequences [14,15,16]. Studies have shown that staying at home increases depression, health anxiety, financial concern, and loneliness [16,17,18]. All limitations and practices have further increased the fear and anxiety of individuals for COVID-19.

High levels of infection, asymptomatic cases, and uncertainty are important characteristics of infectious diseases. Therefore, infectious diseases produce more fear than other diseases. Due to the high and rapid transmission rate and unexpected deaths, people have felt increasing fear of COVID-19. This fear, reduces rational thinking and causes individuals to be stigmatized and excluded in the societal arena.

Psychological and social issues such as fear and anxiety have been ignored since infection control, medicine, and vaccination against COVID-19 came to the fore [19]. Initially, COVID-19 was not taken seriously, and after it was known what the infection would cause, a sense of panic and anxiety emerged [20]. Later, it was understood that people feared being infected themselves and feared infection among their friends or family members [21]. It has been found that those who practice healthcare, teaching, customer service, public transport, security, and certain professions with close customer contact feel more fear of COVID-19 [22]. Therefore, it is thought that this extreme fear will increase the depression, anxiety, and stress levels of these individuals [23]. The chronic illnesses of individuals or the presence of infected relatives and family members increase the fear of COVID-19. If the level of fear of COVID-19 increases, it will be difficult for individuals to behave clearly and rationally while responding to both COVID-19 and other events [21].

### 1.2. The Effect of COVID-19 Fear on Depression, Anxiety, and Stress

Due to the quarantines widely applied and the rapid spreading of information by mass media, a global panic wave was created, producing abnormal behavior in humans [24,25]. As several studies showed, the COVID-19 pandemic has caused physical discomfort due to people needing to stay at home for a long time, working from home, and being inactive [26]. Studies show that COVID-19 may cause long-term psycho-social disorders [26,27]. Negative and inconsistent COVID-19 news, mostly watched by people staying at home, on television, the internet, and social media, make individuals even more worried and can cause an increase in COVID-19-related phobia [24,28,29]. In particular, the prohibitions and quarantines applied to deter the accelerated expansion of COVID-19 modify the lifestyles of individuals and increase anxiety, depression, and stress [30]. COVID-19 fear has a greater impact on anxiety, depression, and stress when individuals are not physically and/or psychologically healthy. In addition to that, witnessing family members or friends becoming infected can intensify the negative impact of that fear. Therefore, COVID-19 fear will increase the psychiatric symptoms in an individual, and further the damage the virus will cause to individuals [31].

Turkish people are very sensitive to traditional processes. Funerals have an important place both religiously and traditionally in Turkish society. The psychological effects of the epidemic will last for many years due to certain negative experiences, such as not being able to fulfill religious and cultural rituals after deaths during the pandemic period [16,32]. Additionally, people lost their jobs [15,33], students were not allowed to go to school, the elderly, who were most affected by the virus, are forced to stay in quarantine for a long time, and married couples and their children have experienced problems [6,34,35] by staying at home for long times, resulting in more psychological problems. These psychological problems have increased tensions in both families and society [36]. During the COVID-19 pandemic, suicide, family and partner violence, and divorce cases have increased several times in many countries [16,37,38,39,40]. Uncertain questions, such as how long the quarantine will last, will there be difficulties in providing necessities, will schools be reopened, how to use health services in case of COVID-19 virus infection, and how the epidemic will recover the economy, occupy everyone’s minds and increase anxiety [41]. The measures taken for COVID-19 particularly affect the poor, immigrants, refugees, internally displaced people, and vulnerable groups who have to supply their labor daily for their livelihoods [42].

**Figure 1:**
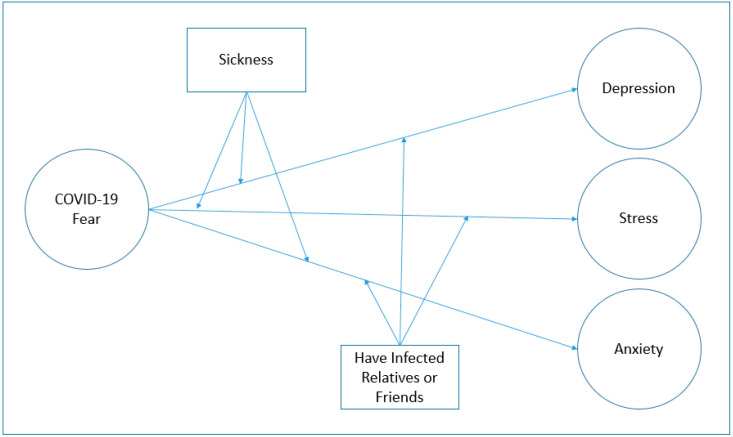
Conceptual Diagram of Model.

An online study was conducted by Odriozola-González et al. [43] with 3550 adult individuals in Spain, a country with many COVID-19 infections. It was understood that 32.4% of the participants had high levels of anxiety, 44.1% presented with depression and 37% showed high levels of stress [43]. Wang et al. [44] evaluated the pandemic’s psychological effects on individuals in a study with 1210 participants living in different cities of China at the end of January and the beginning of February. They stated that 16.5% of the participants showed moderate to severe symptoms of depression, 28.8% showed moderate to severe anxiety symptoms, and 8.1% showed moderate to severe stress symptoms [44]. In another study conducted with 3524 participants, it was seen that individuals between the ages of 18–33 had more severe symptoms of depression, anxiety, and stress during the COVID-19 pandemic; it has been found that older people generally give better psychological responses [45]. A study conducted with students during the COVID-19 period in France found that 24.7% of students had high perceived stress, 16.1% depression, and 27.5% anxiety [46]. In a study conducted on Chinese adolescents (aged 12–18, *n* = 8079), it was found that 37% of the adolescents had anxiety and 43% had depression, with women being the highest risk group [47]. The Figure 1 illustrates conceptual model and our hypothesis 1 is COVID-19 fear increases (i) depression, (ii) anxiety, and (iii) stress.

### 1.3. Moderation Roles of Underling Illness and Having Infected or Dead Relatives and/or Friends 

According to the study done by Kimter [48], psychological resilience and being healthy are important individual characteristics in facing the fear of COVID-19 and the psychological problems caused by this fear. Therefore, those people with strong psychological resilience and a healthy life appear to be less affected by COVID-19 [48]. People’s reactions to extraordinary situations may differ if they have an underlying illness, such that those who are sick in extraordinary situations such as a pandemic become more anxious and depressive. With a similar approach, those who have friends or relatives that have been infected with COVID-19 are thought to be more stressed, anxious, and depressive. Encountering or being contacting with infected people who do not show symptoms further increases this anxiety [49,50]. Many studies have found that people who have been sick frequently before COVID-19 are more likely to be adversely affected by COVID-19 [51,52,53,54,55,56,57]. Some studies have shown that individuals whose family or friends have become ill or have died because of COVID-19 have more stress, anxiety, and depression [50,58,59,60,61,62]. Therefore, based on aforementioned ideas the hypothesis 2 is that having an infected or dead family member, relative, or friend has a moderation effect on COVID-19 fear, which causes (i) depression, (ii) anxiety, and (iii) stress; and hypothesis 3 is that having an underlying illness has a moderation effect on COVID-19 fear, which causes (i) depression, (ii) anxiety, and (iii) stress. 

## 2. Method

### 2.1. Study Design, Participants, and Procedure

Since the purpose of this research was to understand variation in dependent variables at a given time among people in Turkey, we planned a cross-sectional study. We used the process of convenience sampling to assess the sample and used the survey as a medium to gather data. In this research, a quantitative and correlational design was used. Using cross-sectional data, variables were measured at a particular point in time. This research design is appropriate for our goal, as we did not aim to generalize the levels of variables but rather assessed the relationship pattern between variables and to see the frequency of these relationships.

All of the participants were from different regions and cities within Turkey. The individuals were reached online (*n* = 3287). The research started on 8 October 2020, when the COVID-19 cases increased for the second time, and was completed on 26 November 2020, when it peaked. In this period, schools switched to online education, customer restrictions started in the services sector, working hour regulations were introduced in all sectors, and curfews were imposed on evenings and weekends.

The consent of all participants was obtained before applying the questionnaire, and the respondents remained anonymous. All participants were informed about the aims of the research before answering the questionnaire. Technically, participation in the survey was only allowed once. After they started to answer the questionnaire, they were able to terminate it whenever they wanted. Confidentiality and anonymity of data were ensured. The study was carried out in accordance with the Declaration of Helsinki criteria.

### 2.2. Data Analysis

After the data were downloaded from an online survey website, they were transferred to MS Excel for data screening and cleaning. All the analyses were done using IBM SPSS 25. Demographic characteristics of the participants were analyzed using a descriptive analysis. Both categorical classifications and means and standard deviations are represented in the descriptive analysis. Furthermore, a factor analysis of the DASS-21 scale was performed, in order to assure construct validity. To test the hypotheses, depression, anxiety, and stress variables were defined as dependent variables and other variables as independent variables using a multiple regression analysis. We used the PROCESS-Macro [63] plug-in to test moderation hypotheses, and simple slope tests were conducted for two-way interactions [63,64]. Statistical significance was set at α > 90%.

### 2.3. Measures

In this research, age, gender, marital status, education level, infection status, and whether they had any relatives or friends who were infected with or dead because of COVID-19 was asked in the demographic questions section. All of these questions were asked to the participants as categorical variables. Answers to questions regarding participants’ infection status and whether they had any relatives or friends who were infected with or dead because of COVID-19 were obtained with a binary scale (yes = 1, no = 0).

To assess COVID-19 fear, we used the scale Fear of COVID-19 Scale (FCV-19S), which was developed by Ahorsu et al. [65] and adapted to Turkish by Bakioğlu et al. [27]. We used a five-point Likert-type rating scale from 1 = Strongly disagree to 5 = Strongly agree. The Turkish version showed good factorial validity and reliability. Cronbach’s alpha internal consistency was α = 0.88 in the adaptation study [27].

We measured depression, anxiety and stress with the short version of Depression-Anxiety-Stress Scales (DASS-21), which is a shortened version of the DASS developed by Lovibond and Lovibond [66]. The short form of the scale (DASS-21) was developed by Henry and Crawford [67] by using choosing 21 items out of the 42 from the original scale. DASS-21 was adapted to Turkish by Yılmaz et al. [68], and showed sufficient validity and reliability. In the DASS-21 scale, there are 21 questions for the measurement of depression, anxiety, and stress sub-dimensions. The scale uses a four-point Likert type and was coded as 0 = not applicable to me, 1 = slightly applicable to me, 2 = generally applicable to me, and 3 = completely applicable to me.

## 3. Results

### 3.1. Descriptive Analysis

As shown in Table 1, 56.7% of the participants were female and 43.3% were male; moreover, 50.7% were 16–25 years old, 22.4% were 25–40 years old, 25.5% were 40–65 years old, and 1.4% were 65–96 years old (AgeMean = 31.75 ± 13.77). In total, 60.6% of the participants were single and 39.4% were married (Marital Status Mean = 1.39 ± 0.49). Participants’ education levels were as follows: 0.2% did not graduate from any school, while 2.3% were elementary graduates and 2.1% were middle school graduates. In addition, 11.4% of the participants were high school graduates, 66.3% graduated from university and 17.8% were masters or PhD graduates (Education Mean = 4.95 ± 0.78). When the participants were asked whether they had underlying illnesses, 69.6% answered yes and 30.4% answered no (Sickness Mean = 0.30 ± 0.46). Among the participants, the percentage of people who had family or friends that were infected with or had died from COVID-19 was 62.3%, and the percentage of those who had no family or friends that were infected with or had died from COVID-19 was 37.7% (Mean = 0.62 ± 0.48).

### 3.2. Correlations between Variables

The correlations among study variables including demographics, means, and standard deviations are shown in Table 2. According to Table 2, the COVID-19 fear level was higher in females (*r* = 0.236, *p* < 0.01), lower as age increased (*r* = −0.183, *p* < 0.01), and decreased in married individuals (*r* = −0.158, *p* < 0.01). The COVID-19 fear level decreased as the education level increased (*r*= −0.053, *p* < 0.01) and increased with the presence of underlying illness (*r* = 0.068, *p* < 0.01). It was determined that as the COVID-19 fear increased, depression, anxiety, and stress levels (*r* = 0.354, *r* = 0.514, *r* = 0.365, respectively, *p* < 0.01) increased. Depression, anxiety, and stress levels in women (*r* = 0.177, *r* = 0.187, *r* = 0.242, respectively, *p* < 0.01), in singles (*r* = −0.296, *r* = −0.190, *r* = −0.286, respectively, *p* < 0.01), in young people (*r* = −0.309, *r* = 0.203, *r* = −0.309, respectively, *p* < 0.01), and in those with underlying illness (*r* = 0.082, *r* = 0.161, *r* = 0.116, respectively, *p* < 0.01) was determined to be statistically higher. Anxiety and stress were lower in educated people (*r* = −0.067, *r* = −0.054, respectively, *p* < 0.01) and higher in those whose friends or family had been infected with or died from COVID-19 (*r* = 0.079, *r* = 0.049, respectively, *p* < 0.01).

### 3.3. Hypothesis Testing

Hypothesis 1 suggested that fear of COVID-19 increases depression, anxiety, and stress. To test this hypothesis, three separate hierarchical regression analyses were performed. Since some of the demographic variables, such as gender, age, marital status, education, and underlying illness, had significant correlations with the outcome variables anxiety, stress, and depression, they were used as control variables in all models. The direct effects were included in Model 2 in each analysis. In the analysis for depression, seen in Table 3, Model 2 was found to be significant (F = 124.34, R^2^ = 0.21, ΔR^2^ = 0.10, *p* < 0.001); COVID-19 fear had a significantly positive effect on depression (B = 0.25, *p* < 0.001). In the analysis for anxiety, shown in Table 4, Model 2 was found to be significant (F = 207.56, R^2^ = 0.31, ΔR^2^ = 0.25, *p* < 0.001); COVID-19 fear had a significantly positive effect on anxiety (B = 0.27, *p* < 0.001). As seen in Table 5, in the analysis for stress, Model 2 was found to be significant (F = 137.41, R^2^ = 0.23, ΔR^2^ = 0.11, *p* < 0.001); COVID-19 fear had a significantly positive effect on stress (B = 0.24, *p* < 0.001). Therefore, hypothesis 1 was accepted.

Hypothesis 2 suggested that having an infected or dead family member, relative, or friend moderates the effect of COVID-19 fear, which can cause (i) depression, (ii) anxiety, and (iii) stress. Hypothesis 3 suggested that having an underlying illness moderates the effect of COVID-19 fear, which can cause (i) depression, (ii) anxiety, and (iii) stress. In order to test hypothesis 2 and 3, the effects of the interaction terms on the dependent variables (depression, anxiety, and stress) are shown in Model 3, see Table 3, Table 4, and Table 5. Moderation variables (underlying illness, infected relatives or friends) were both added to the existing variables in each Model 3. Model 3 in Table 3 (F = 100.96, R^2^ = 0.22, ΔR^2^ = 0.01, *p* < 0.001), Table 4, (F = 174.94, R^2^ = 33, ΔR^2^ = 0.02, *p* <.001), and Table 5 (F = 110.26, R^2^ = 0.23, ΔR^2^ = 0.01, *p* < 0.001) were found to be statistically significant. The interaction effect of the COVID-19 fear variable and the variable of infected or dead relatives or friends on depression, anxiety, and stress (B = 0.05, *p* < 0.01; B = 0.05, *p* < 0.001; B = 0.06, *p* < 0.001, respectively) was statistically significant. The interaction effect of the COVID-19 fear variable and underlying illness variables on depression, anxiety, and stress (B = 0.09, *p* < 0.001; B = 0.10, *p* < 0.001; B = 0.06, *p* < 0.05, respectively) was statistically significant. Therefore, hypothesis 2 and hypothesis 3 were accepted.

**Figure 2:**
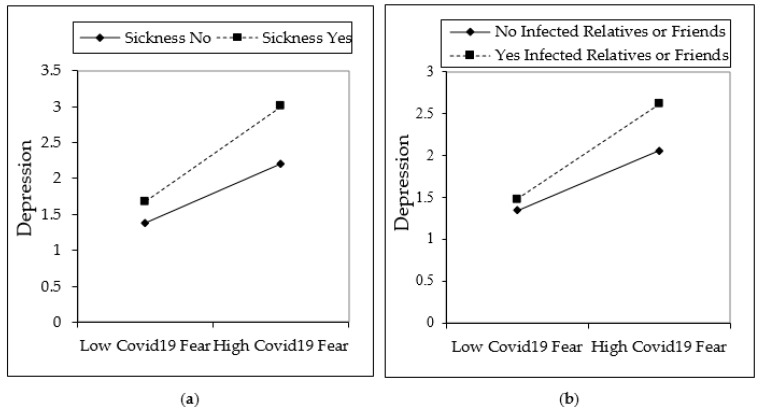
Interaction effects on depression. Moderators of underlying sickness is depicted in (**a**), having infected relatives or friends is depicted in (**b**).

**Figure 3:**
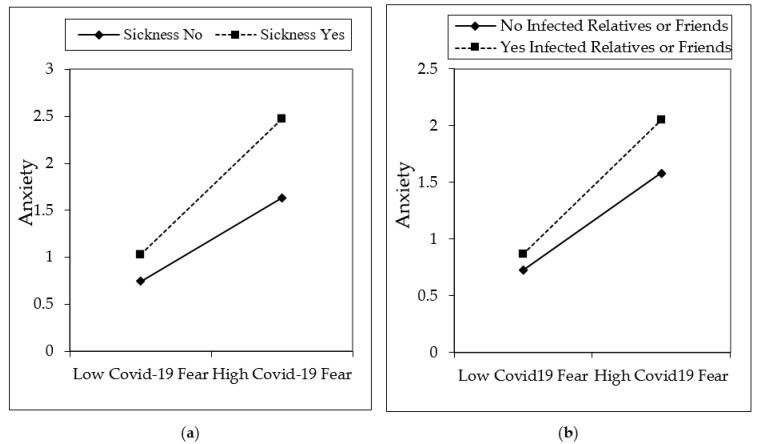
Interaction effects on anxiety. Moderators of underlying sickness is depicted in (**a**), having infected relatives or friends is depicted in (**b**).

**Figure 4:**
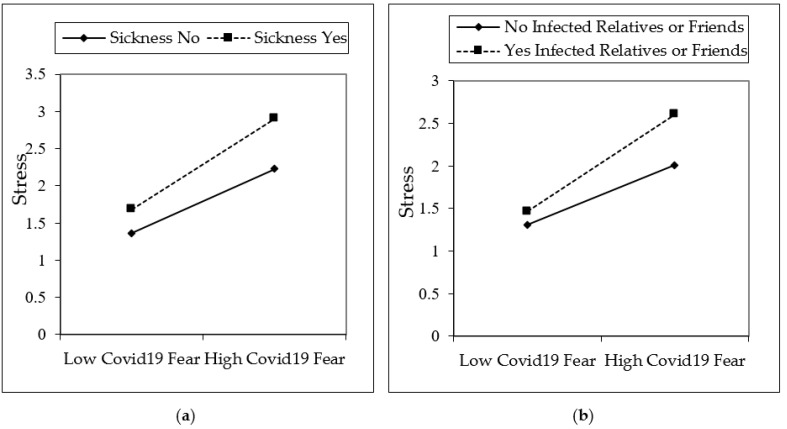
Interaction effects on stress. Moderators of underlying sickness is depicted in (**a**), having infected relatives or friends is depicted in (**b**).

Figure 2, Figure 3 and Figure 4 are shown in two groups as moderation effects a and b. The moderation effect of individuals’ underlying illness situations on the effect of COVID-19 fear, and its effect on depression, anxiety, and stress, is demonstrated by (a). The moderation effect of having an infected or dead family member, relative, or friend of individuals on COVID-19 fear, and its effect on depression, anxiety, and stress is demonstrated by (b). The results are illustrated in Figure 2, Figure 3, and Figure 4.

## 4. Discussion

This study was conducted to understand whether fear of COVID-19 has an effect on psychological results such as depression, anxiety, and stress. While analyzing, questions such as the participants’ age, gender, marital status, education level, and health status were used as control variables. All three hypotheses of the study were fully supported. Hypothesis 1, which argues that COVID-19 fear increases (i) depression, (ii) anxiety, and (iii) stress, was tested and confirmed in this study. COVID-19 fear was independently associated with those psychological outcomes. Additionally, hypothesis 2, which argues that having an infected or dead family member, relative, or friend has a moderation effect on COVID-19 fear, which causes (i) depression, (ii) anxiety, and (iii) stress, was fully confirmed. Hypothesis 3, which argues that having an underlying illness has a moderation effect on COVID-19 fear, which causes (i) depression, (ii) anxiety, and (iii) stress, was accepted. The theory of this study was based on the approach explained by Işıklı [7]. According to Işıklı, people will confront the psychological consequences of COVID-19 fear, and some risks, such as underlying illness, fear of virus transmission, or fear of dying from the virus, will increase these psychological consequences more.

As predicted, during extraordinary periods such as the pandemic, people change their previous lifestyles, live in more closed areas, are left alone, are affected by negative news, and feel fear and anxiety due to quarantines and curfews [30]. The negative effects of extraordinary situations are felt less by individuals with high psychological strength [48]. Disadvantaged and fragile segments of society are affected much more negatively. Today, COVID-19 has spread worldwide and has damaged societies both physically and psychologically [26,69]. As a kind of disaster, the COVID-19 pandemic, just as previous pandemics, has caused mass deaths and great suffering [20]. Therefore, these problems during the pandemic have negative reflections on both the individual and society. Thus, the long-term damage caused by the COVID-19 virus will be the psychological disorders caused by the COVID-19 fear [31].

In many studies, it has been found that the distress during the pandemic increases the fear of COVID-19, and this rising fear increases depression, anxiety, and stress symptoms of individuals. Fear of the unknown increases anxiety levels in healthy individuals and in those who already have health problems [70]. In Ahorsu’s study on Iranians, it was seen that COVID-19 fear exacerbated psychiatric symptoms such as depression and anxiety [65]. In a study conducted with 1879 individuals in the early stage of the pandemic in the Philippines, the DASS-21 scale was used, and increases in individuals’ depression, anxiety, and stress levels were found [71]. In a literature study, it was found that in addition to the general population experiencing significant psychological problems, depression, anxiety, and stress increased, especially among healthcare workers, students, service sector workers, women, those in quarantine, and those exposed to misinformation [72]. The current study found that COVID-19 fear raises individuals’ depression, anxiety, and stress levels above the accepted level, consistent with many other studies [43,44,45,46,47].

The fear of COVID-19 is expected to increase if individuals are in poor health or have an underlying illness. In this study, it was found that COVID-19 fear increases if individuals are sick or in poor health. It has even been observed that many people who are sick do not go to the hospital for examination due to the fear of COVID-19 infection. Since sick people are more affected by COVID-19, those who are sick have more COVID-19 fear levels [51,52,73]. Similarly, more COVID-19 fear is felt when individuals have relatives or friends who have been infected by or died from COVID-19. Hence, this fear also increases individuals’ states of depression, anxiety, and stress [58,59,61,62]. An interaction effect can be seen from Figure 2, Figure 3 and Figure 4, which show the moderation analysis. Hypotheses 2 and 3 of the study were confirmed in this study, which coincides with similar findings in the literature.

During pandemic periods, individuals’ fear of pandemics is extremely high due to people being influenced by negative news, quarantine, and curfews, and experiencing infection or death among their family, relatives, and friends. This study’s theory is that fear of pandemics disrupts people’s psychology and that the psychology of those who had an underlying illness before the pandemic or had family or friends who were infected or had died is much more impaired. This holistic approach has not been seen in the models of other studies before. Therefore, this study shows that it will be beneficial to produce special approaches for those who have the disease before and those most affected by the pandemic among the people affected by COVID-19 fear that emerged during the pandemic. In addition to these groups, it is understood from both the literature and this study that women, young people, singles, and unemployed people are also affected negatively. Due to the lack of sufficient academic studies on this subject in Turkey, this study’s results are expected to be leading for academics and policymakers. In this sense, it is necessary to implement different applications for different groups who are disadvantaged and fragile during the pandemic period.

## 5. Limitations and Suggestions for Future Studies

The sample size of this study and its representation of different groups are its strengths. However, the study’s weaknesses are that data were collected within a certain period, the possibility of self-reporting of individuals causing potential bias, and the high number of students and low numbers of elderly among the participants. In the future, it is necessary to carry out studies that focus on certain groups and that show comparisons between periods to reach more specific findings. Additionally, we did not know the stress, anxiety, and depression levels of participants before the COVID-19 pandemic, making it difficult to interpret effects, as prior levels may be confounding. However, since COVID-19 appeared very suddenly, it was impossible to conduct pre-test beforehand.

## 6. Conclusions

This study provides preliminary evidence that the negative psychological impact of the COVID-19 outbreak affects those in certain situations more. In particular, current results should be taken into account when developing evidence-based policies and practices. With this study, it was understood that the pandemic had not only physical consequences but also psychological consequences. In fact, these psychological consequences are predicted to be long-term, unless action is taken. This preliminary work provides academicians and policymakers a glimpse of what may happen in a pandemic, and which parts of societies are the most affected and vulnerable. Moreover, in the case of the pandemic, this study shows what kind of strategies can be implemented in the years to come to those who are negatively affected by the psychological consequences of pandemic fear in Turkey. These practices should include an approach in the form of support strategies specific to each group.

## Figures and Tables

**Table 1:** Descriptive Statistics.

		*f*	%	M	SD
Gender				1.57	0.50
	Female	1863	56.7		
	Male	1424	43.3		
Age				31.78	13.77
	16–25	1667	50.7		
	25–40	737	22.4		
	40–65	837	25.5		
	65–96	46	1.4		
Marital Status				1.39	0.49
	Single	1993	60.6		
	Married	1294	39.4		
Education Level				4.95	0.78
	No Graduation	7	0.2		
	Elementary	74	2.3		
	Middle School	70	2.1		
	High School	374	11.4		
	University	2178	66.3		
	Masters or PhD	584	17.8		
Underlying Illness				0.30	0.46
	I Have an Underlying Illness	2288	69.6		
	I Have no Underlying Illness	999	30.4		
Infected Relatives or Friends				0.62	0.48
	No	1240	37.7		
	Yes	2047	62.3		
Total		3287	100%		

**Table 2:** Means, Standard Deviations, Reliabilities, and Correlations.

No		1	2	3	4	5	6	7	8	9	10
1	Gender (1 = m. 2 = f.)										
2	Age	−0.442 **									
3	Marital St. 1–2 = No–Yes	−0.408 **	0.752 **								
4	Education	−0.095 **	−0.015	−0.029							
5	COVID-19 Fear	0.236 **	−0.183 **	−0.158 **	−0.053 **	(0.87)					
6	Underlying Illness, 0–1 = No–Yes	−0.02	0.233 **	0.134 **	−0.087 **	0.068 **					
7	Infected Relatives or Friends, 0–1 = No–Yes	−0.023	−0.001	0.053 **	0.024	0.021	0.080 **				
8	Stress	0.242 **	−0.309 **	−0.286 **	−0.054 **	0.365 **	0.116 **	0.049 **	(0.89)		
9	Anxiety	0.187 **	−0.203 **	−0.190 **	−0.067 **	0.514 **	0.161 **	0.079 **	0.564 **	(.85)	
10	Depression	0.177 **	−0.31 **	−0.296 **	−0.027	0.35 **	0.08 **	0.03	0.73**	0.56 **	(0.91)
	Mean	1.57	31.78	1.39	4.95	2.48	0.30	0.62	0.72	0.32	0.55
	Sd.	0.49	13.77	0.49	0.78	0.83	0.46	0.48	0.70	0.47	0.69

** Correlation is significant at the 0.01 level (two-tailed). Diagonal values in parentheses represent the Cronbach alpha.

**Table 3:** Main and interaction effects on depression.

Variable	Model 1			Model 2			Model 3		
	B	SE	*p*	B	SE	*p*	B	SE	*p*
Constant	1.20	0.10	<0.001	−0.57	0.10	<0.001	0.92	0.13	<0.001
Gender	0.05	0.03	0.07	−0.05	0.03	0.07	−0.04	0.03	0.08
Age	−0.01	0.00	<0.001	−0.01	0.00	<0.001	−0.01	0.00	<0.001
Marital Status, 1 = single 2 = married	−0.20	0.04	<0.001	−0.18	0.03	<0.001	−0.19	0.03	<0.001
Education	−0.03	0.02	0.06	−0.01	0.01	0.47	−0.01	0.01	0.51
COVID-19 Fear				0.25	0.01	<0.001	0.10	0.04	0.01
Underlying Illness (0 = No 1 = Yes)				0.19	0.03	<0.001	0.18	0.02	<0.001
Infected Relatives or				0.03	0.02	0.21	0.03	0.02	0.26
Friends (0 = No 1 = Yes)									
COVID-19 Fear × Infected Rel. or Fr.							0.05	0.02	0.00
COVID-19 Fear × Underlying Illness							0.09	0.02	<0.001
F		98.14			124.34			100.96	
*p*		<0.001			<0.001			<0.001	
R2		0.11			0.21			0.22	
R2 Change									

**Table 4:** Main and interaction effects on anxiety.

Variables	Model 1			Model 2			Model 3		
	B	SE	*p*	B	SE	*p*	B	SE	*p*
Constant	0.55	0.07	<0.001	−0.14	0.07	0.03	0.20	0.08	0.02
Gender	0.10	0.02	<0.001	0.01	0.02	0.72	0.01	0.02	0.63
Age	0.00	0.00	<0.001	0.00	0.00	<0.001	0.00	0.00	<0.001
Marital Status, 1 = single 2 = married	−0.07	0.03	0.01	−0.05	0.02	0.01	−0.06	0.02	0.01
Education	−0.04	0.01	<0.001	−0.02	0.01	0.03	−0.02	0.01	0.03
COVID-19 Fear				0.27	0.01	<0.001	0.13	0.02	<0.001
Underlying Illness (0 = No 1 = Yes)				0.16	0.02	<0.001	0.15	0.02	<0.001
Have Infected Relatives or Friends (0 = No 1 = Yes)				0.06	0.01	<0.001	0.06	0.01	<0.001
COVID-19 Fear × Infected Rel. or Fr.							0.05	0.01	<0.001
COVID-19 Fear × Underlying Illness							0.10	0.02	<0.001
F		50.87			207.56			174.94	
*p*		<0.001			<0.001			<0.001	
R^2^		0.06			0.31			0.33	
R^2^ Change					0.25			0.02	

**Table 5:** Main and Interaction Effects on Stress.

Variable	Model 1			Model 2			Model 3		
	B	SE	*p*	B	SE	*p*	B	SE	*p*
Constant	1.19	0.11	<0.001	0.54	0.10	<0.001	0.89	0.13	<0.001
Gender	0.16	0.03	<0.001	0.07	0.03	0.01	0.07	0.03	<0.001
Age	−0.01	0.00	<0.001	−0.01	0.00	<0.001	−0.01	0.00	<0.001
Marital Status, 1 = single 2 = married	−0.15	0.04	<0.001	−0.13	0.03	<0.001	−0.13	0.03	<0.001
Education	−0.04	0.02	0.00	−0.03	0.01	0.08	−0.02	0.01	0.09
COVID-19 Fear				0.24	0.01	<0.001	0.10	0.04	0.01
Underlying Illness (0 = No 1 = Yes)				0.23	0.02	<0.001	0.22	0.02	<0.001
Have Infected Relatives or Friends (0 = No 1 = Yes)				0.05	0.02	0.02	0.05	0.02	0.02
COVID-19 Fear × Infected Rel. or Fr.							0.06	0.02	<0.001
COVID-19 Fear × Underlying Illness							0.06	0.02	0.01
F		107.71			137.41			110.26	
*p*		<0.001			<0.001			<0.001	
R2		0.12			0.23			0.23	
R2 Change					0.11			0.01	

## Data Availability

The data presented in this study are available on request from the corresponding author. The data are not publicly available due to that they are a part of a developing dataset which will be used in the future for different studies.
